# Immune‐related gene *TM4SF18* could promote the metastasis of gastric cancer cells and predict the prognosis of gastric cancer patients

**DOI:** 10.1002/1878-0261.13321

**Published:** 2022-10-20

**Authors:** Xinyue Qin, Yinhao Chen, Shuo Ma, Lei Shen, Shaoqing Ju

**Affiliations:** ^1^ Department of Laboratory Medicine Affiliated Hospital of Nantong University, Medical School of Nantong University China; ^2^ Research Center of Clinical Medicine Affiliated Hospital of Nantong University China; ^3^ Medical School of Southeast University Nanjing China

**Keywords:** biomarker, gastric cancer, prognosis, *TM4SF18*

## Abstract

Gastric cancer (GC) is one of the most common malignancies in the world, and the search for better markers has become one of the challenges today. It has been found that the L6 superfamily regulates the biological functions of numerous tumors, but transmembrane 4 L six family member 18 (*TM4SF18*) has been rarely reported. We found that *TM4SF18* expression is upregulated in GC tissues and cells, which can be effectively diagnosed and dynamically monitored to assess the prognosis of GC patients. Furthermore, knockdown of *TM4SF18* effectively inhibited proliferation, migration, and invasion of GC cells, and affected the epithelial‐mesenchymal transition process. *TM4SF18* was found to be an independent prognostic factor for GC by univariate and multifactorial Cox analyses as well as by establishing nomogram plots. In addition, in *TM4SF18* and immune correlation analysis, *TM4SF18* expression levels were found to be negatively correlated with most immune cell marker genes and associated with numerous immune cells and immune pathways, resulting in less benefit from treatment with immune checkpoint inhibitors. In summary, we found that *TM4SF18* is a promising GC biomarker that promotes the proliferation, migration, and invasion abilities of GC cells, and is associated with immune response.

## Graphical abstract

Gastric cancer (GC) is one of the most common malignancies in the world and there is a need to find better markers. We found that *TM4SF18* expression is upregulated in GC tissues and cells, regulates GC cell function, and correlates with the immune microenvironment, and *TM4SF18* could act as a good biomarker in GC.

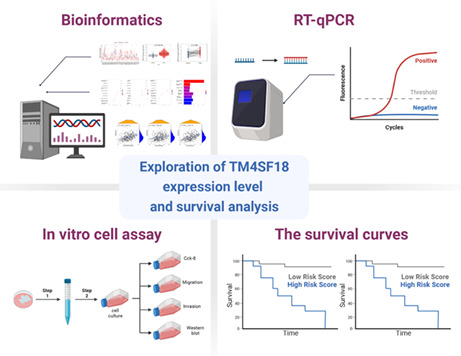

**Abbreviations**
aDCs: activated dendritic cells
AJCC: American Joint Committee on Cancer
AUC: area under curve
CCK‐8: Cell Counting Kit‐8
CCR: chemokine receptors
CI: confidence interval
EMT: epithelial‐mesenchymal transition
ESTIMATE: Estimation of Stromal and Immune Cells in Malignant Tumor Tissues Using Expression Data
GC: gastric cancer
GES‐1: gastric epithelial cells
GO: Gene Ontology
GSEA: gene set enrichment analysis
HNSC: head and neck squamous cell carcinoma
ICI: immune checkpoint inhibitor
KEGG: Kyoto Encyclopedia of Genes and Genomes
KICH: kidney chromophobe
LUSC: lung squamous cell carcinoma
MSI: microsatellite instability
N‐cad: N‐cadherin
ROC: receiver operating characteristic
STAD: stomach adenocarcinoma
TCGA: The Cancer Genome Atlas
TIDE: tumor immune dysfunction and exclusion
TIL: tumor infiltrating lymphocytes
TM4SF18: transmembrane 4 L six family member 18
TMB: tumor mutational burden
TME: tumor microenvironment

## 1 Introduction

Gastric cancer (GC) is one of the most common tumors in the world and has the fifth highest incidence rate worldwide. The 2020 Global Cancer Statistics shows that there are nearly 1 million new cases of GC each year [1, 2]. The main causes of GC include *Helicobacter pylori* infection, precancerous lesions, and genetic factors [1, 3]. In most cases, GC can metastasize through the lymph nodes to adjacent tissues and among them and produce more cancer cells through the blood [4]. Surgical treatment and adjuvant therapy remain the primary treatment for patients with GC [5]. Although there have been great advances in the diagnosis and treatment of GC, the prognosis of patients with GC remains poor owing to tumor recurrence and metastasis [6, 7]. Therefore, the treatment of GC remains a considerable challenge, and more approaches are needed to optimize treatment and improve prognosis. In recent years, with the continuous development of high‐throughput sequencing technology and bioinformatics analysis, an increasing number of genes have been identified. However, most of the differential genes do not have relatively good specificity and sensitivity; therefore, the search for reliable biomarkers is crucial for the diagnosis and prognosis of GC.

The L6 superfamily is a substance with four transmembrane structural domains and consists of six members (*TM4SF1*, *TM4SF4*, *TM4SF5*, *TM4SF18*, *TM4SF19*, and *TM4SF20*) [8]. Owing to their structural similarity, the members of the L6 family were initially thought to be Tetraspanins, but as research continued, it was discovered that the family did not belong to the Tetraspanins but formed its own family of proteins. This family can regulate cell invasion, migration, epithelial‐mesenchymal transition (EMT), adhesion, and cell growth through interactions with non‐covalent molecules of integrins [9, 10, 11, 12]. In recent years, both *TM4SF18* and *TM4SF1* are overexpressed in pancreatic cancer. *TM4SF1* has been shown to promote metastasis and cell motility by inducing inactivation and regulating matrix metalloproteinase activity but not cell growth [13]. *TM4SF18* has been found to promote cell growth but not regulate migration in pancreatic cancer cells [14]. The above suggests that *TM4SF18* can regulate the progression of some tumors, but the regulatory mechanism of *TM4SF18* in GC is currently unexplored.

In this study, we evaluated the diagnostic and prognostic values of the *TM4SF18* gene in human GC by analyzing data from The Cancer Genome Atlas (TCGA) dataset. It was found that *TM4SF18* may regulate the signaling pathway of GC. In addition, knockdown of *TM4SF18* was demonstrated to effectively inhibit the proliferation, migration, and invasion abilities of GC cells by *in vitro* cellular assays. Finally, we explored the relationship between *TM4SF18* expression, tumor immune infiltration, and tumor microenvironment (TME). This provides a more theoretical basis for *TM4SF18* as a GC biomarker and potential therapeutic target.

## 2 Materials and methods

### 2.1 The Cancer Genome Atlas‐based data collection

All pan‐cancer data (*N* = 10 535) were obtained from the UCSC Xena website (http://xena.ucsc.edu/). Data from the TCGA database of 375 GC tissues and 32 GC adjacent tissues were downloaded. The data from the TCGA database were used to compare the differences in *TM4SF18* expression in pan‐cancer and also to compare the differences in *TM4SF18* expression in GC tissues and normal tissues adjacent to GC.

### 2.2 Tissue specimens

Forty GC tissues and 40 adjacent tissues of GC were obtained from the Department of Pathology, Nantong University Hospital (which contained 23 pairs of GC tissues and paired adjacent normal tissues). All obtained tissues were immediately stored in a −80 °C refrigerator. Pathological data were classified according to the American Joint Committee on Cancer (AJCC) 8th edition clinical practice guidelines for GC. The study methodologies conformed to the standards set by the Declaration of Helsinki. The Ethics Committee of Affiliated Hospital of Nantong University (Ethics Review Report No. 2018‐L055) approved the study. All participants gave informed written consent before the clinical trial and gave consent to publish.

### 2.3 Cell culture

Human GC cell lines (SGC‐7901, MKN‐45, MKN‐1, AGS, and BGC‐823) and gastric epithelial cells (GES‐1) were purchased from the Shanghai Cell Bank, Chinese Academy of Sciences. The aforementioned cells were cultured in RPMI‐1640 medium (Corning, New York, NY, USA). *TM4SF18* was knocked down in MKN‐45 and SGC‐7901 cells for subsequent *in vitro* cell experiments.

### 2.4 Total RNA extraction and RT‐qPCR experiments

Total RNA was extracted from GC tissues and cells using FastPure^®^ Cell/Tissue Total RNA Isolation Kit V2 (Vazyme Biotech Co., Ltd., Nanjing, China) kit. Total RNA extracted from GC tissues and cells was reverse transcribed into cDNA using a reverse transcription kit (Thermo Fisher Scientific, Waltham, MA, USA). Thereafter, the cDNA was diluted fivefold for subsequent experiments. The PCR procedure was performed using Q5 (Thermo Fisher Scientific) (total system 20 μL denaturation 95 °C, 10 s; annealing 60 °C, 30 s; extension 72 °C, 30 s; 45 cycles; all molecular internal references were GD rRNA). The primer sequences were *TM4SF18*‐F: TCTGGATACTGCCTGGTCATCTCTG, *TM4SF18‐*R: AAAAGCATACTCCCAGCCATCAAGG; GAPDH‐F: TCCCATCACCATCTTCCAGG, and GAPDH‐R: GATGACCCTTTTGGCTCCC.

### 2.5 Western blot

Proteins were extracted from GC tissues using RIPA lysate (SolarBio Life Science, Beijing, China). Tissue protein lysis products were electrolyzed using 12% SDS/PAGE (Shanghai EpiZyme Biotechnology, Shanghai, China). Thereafter, they were transferred to a polyvinylidene–fluoride membrane (Millipore, Billerica, MA, USA). Immunoblots were visualized by an ECL detection system (Vazyme Biotech Co., Ltd.). Antibodies against GADPH were used as controls. Antibodies against GADPH, N‐cadherin (N‐cad), and vimentin were obtained from Cell Signaling Technology, Danvers, MA, USA. All three antibodies were diluted at 1 : 1000.

### 2.6 Immunohistochemistry assay

Three pairs of GC tissues and paired GC adjacent tissues were fixed in 4% paraformaldehyde and subsequently paraffin‐embedded. The paraffin sections were sectioned, dewaxed, hydrated, antigenically repaired, closed with 10% goat serum, incubated with anti‐*TM4SF18* antibody overnight at 4 °C, and then labeled with secondary antibody at room temperature for 30 min. The specimens were labeled with DAB dye (DAKO, Copenhagen, Denmark) for 10 min at room temperature and restained. Finally, images were collected for analysis. Antibodies against *TM4SF18* were obtained from Absin (Shanghai, China). Antibody dilution was 1 : 20.

### 2.7 Functional enrichment analysis of *TM4SF18*

Kyoto Encyclopedia of Genes and Genomes (KEGG) and Gene Ontology (GO) analyses were performed on *TM4SF18*, and this work revealed the function of *TM4SF18* in biological processes, molecular functions, and pathway enrichment results. The “ggplot2,” “enrich plot,” and “cluster profiler” packages in r were used to perform GO and KEGG analyses. Statistical significance was set at *P* < 0.05 and *q* < 0.05.

### 2.8 Gene set enrichment analysis pathway enrichment analysis

To investigate the role of *TM4SF18* expression on biological processes in GC, GC patients were divided into high‐ and low‐expression groups based on the median *TM4SF18* expression (cutoff = 2.929). Pathway enrichment analysis of the annotated gene set (c2.cp.kegg.v7.2.symbols.gmt) was performed using Gene Set Enrichment Analysis (GSEA) version 4.1.0 downloaded from the Broad Institute. The effect of synergistic changes in the genes in this gene set on phenotypic changes was determined by correlating the known functional gene set with the *TM4SF18* gene expression matrix. The enrichment score calculated for each gene subset was normalized according to the size of the gene set to obtain a normalized enrichment score (adjusted *P* value < 0.05 for screening conditions).

### 2.9 Cell Counting Kit‐8 assay

Cell Counting Kit‐8 (CCK‐8) assay was used to detect cell proliferation ability. Three thousand transfected cells were inoculated in 96‐well plates, 10 μL of CCK‐8 reagent (Dojindo, Kumamoto, Japan) was added after walling, and the absorbance of cells at 450 and 630 nm was measured every 24 h for 5 days.

### 2.10 Transwell assay

Transwell assay was used to detect the migration and invasion abilities of cells. In Transwell chambers (Corning), 5 × 10^4^ and 8 × 10^4^ cells were inoculated. Matrix gel (BD Biosciences, San Jose, CA, USA) was added to the chambers 24 h in advance before the invasion assay was performed. Both experiments required the addition of serum‐free base gel to the upper chamber and a medium containing 20% serum to the lower chamber. After incubation for 24–48 h, the cells were fixed using paraformaldehyde, stained with crystal violet, and photographed.

### 2.11 Construction of nomogram plots

The predictive power of nomogram plots and other predictors (age, TNM stage, T stage, N stage, and M stage) for 1‐, 3‐, and 5‐year OS was established. A correction curve based on the Hosmer–Lemeshow test was applied to illustrate the concordance between actual and model predicted outcomes.

### 2.12 Immune infiltration analysis

Estimation of Stromal and Immune Cells in Malignant Tumor Tissues Using Expression Data (ESTIMATE) is a tool used to calculate and estimate the extent of stromal cell and immune cell infiltration in malignant tissues. Calculated immune scores were downloaded from the ESTIMATE database for the TCGA cohort. Patients with GC were divided into two groups based on the median immune score.

The TIMER (https://cistrome.shinyapps.io/timer/) database allows for a comprehensive analysis of the level of infiltration of different immune cells. In this study, *TM4SF18* expression was assessed in several cancer types by the “ggpubr” package. The correlation between *TM4SF18* and immune cell infiltration in GC was analyzed in TIMER. The “Gene” module allows the study of the relationship between *TM4SF18* expression and the level of immune cell infiltration (B cells, CD8^+^ T cells, CD4^+^ T cells, neutrophils, macrophages, and dendritic cells) using the TCGA database. The relationship between *TM4SF18* expression and different sets of gene markers in immune cells was also investigated by the “correlation” module. Spearman's correlation and statistical significance were used to assess the relevance of *TM4SF18* expression to immune infiltration.

### 2.13 Prediction of immunotherapeutic response

The r package maftools was used to evaluate and summarize mutation data. Tumor mutational burden (TMB) was measured according to tumor‐specific mutation genes [15]. The Tumor Immune Dysfunction and Exclusion (TIDE) algorithm was used to predict the likelihood of immune therapy response [16].

### 2.14 Statistical analysis

Survival rates were calculated using the Kaplan–Meier method, and log‐rank tests were used for the significance of differences. Univariate and multifactorial analyses were performed using the Cox proportional risk model with stepwise regression (LRForward). The differences between the two groups were compared using *t* tests or the Mann–Whitney tests. Patients were divided into the *TM4SF18* high‐expression group and *TM4SF18* low‐expression group according to the median *TM4SF18* expression (2.929). *P* < 0.05 was considered statistically significant.

## 3 Results

### 3.1 Expression of *TM4SF18* and its diagnostic value

**Fig. 1:**
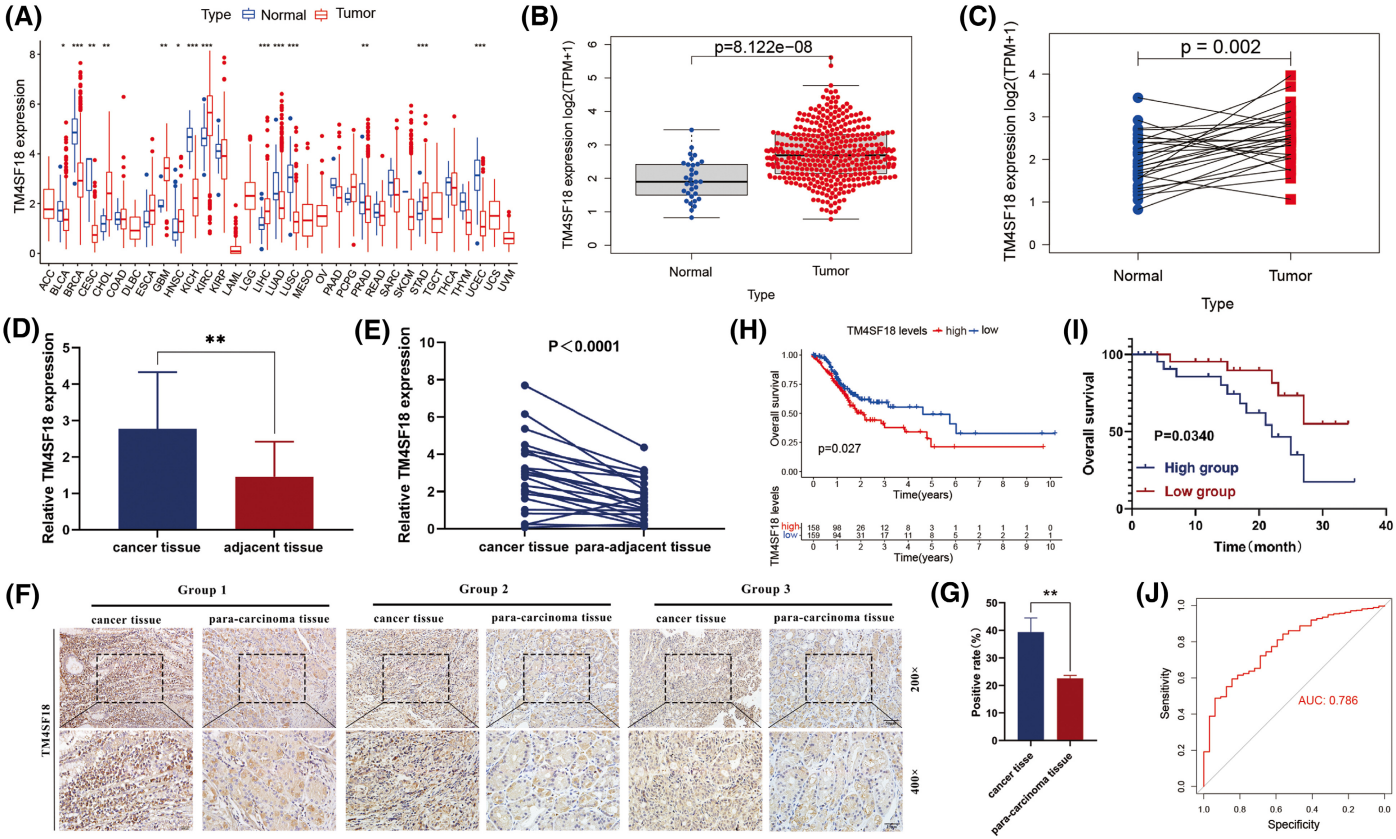
Expression of *TM4SF18* and its diagnostic value. (A) Expression levels of *TM4SF18* in human pan‐cancer tissues (*N* = 10 535, data from the UCSC Xena website, http://xena.ucsc.edu/). (B, C) Expression of *TM4SF18* gene in 375 GC tissues and 32 GC adjacent tissue samples in the TCGA database. (D) RT‐qPCR experiments to verify the expression of *TM4SF18* in 40 GC tissues and 40 adjacent tissues. (E) RT‐qPCR experiments were performed to verify the expression of *TM4SF18* in 23 pairs of GC tissues and adjacent normal tissues. (F, G) Immunohistochemical validation of *TM4SF18* expression in three pairs of GC tissues (magnification ×200 and ×400, respectively; scale bars are 50 and 25 μm). (H) Relationship between *TM4SF18* expression and overall patient survival in 317 GC patients from the TCGA database (excluding patients with missing clinical data and unknown clinical data). (I) Kaplan–Meier survival curves to validate the prognostic value of *TM4SF18* (*N* = 20). (J) ROC curves of *TM4SF18* expression in 375 GC tissues and 32 adjacent normal tissues (without excluding any data). The significance was determined by using Wilcoxon test for A–C, Mann–Whitney test for D, *t* test for E and G, and log‐rank test for H and I. All error bars represent SD. ***P < 0.001, **P < 0.01, *P < 0.05.

To investigate the diagnostic value of *TM4SF18* in GC, we first analyzed the expression of *TM4SF18* in pan‐cancer using the TCGA database and found significant differences in the expression levels of *TM4SF18* in numerous tumors, including bladder urothelial carcinoma, breast invasive carcinoma, cervical squamous cell carcinoma, and endocervical adenocarcinoma, cholangiocarcinoma, glioblastoma multiforme, head and neck squamous cell carcinoma (HNSC), kidney chromophobe (KICH), kidney renal clear cell carcinoma, liver hepatocellular carcinoma, lung adenocarcinoma, lung squamous cell carcinoma (LUSC), prostate adenocarcinoma, stomach adenocarcinoma (STAD), and uterine corpus endometrial carcinoma (Fig. 1A). We downloaded the information of 375 GC tissues and 32 GC adjacent tissue samples from the TCGA database, performed *TM4SF18* expression level analysis, and found that the expression level of *TM4SF18* was significantly upregulated in GC tissues compared with GC adjacent tissues (Fig. 1B,C). Subsequently, we examined the expression levels of *TM4SF18* in 23 pairs of GC tissues and paired GC adjacent tissues by RT‐qPCR assay and found that the expression levels of *TM4SF18* were significantly upregulated in GC tissues (Fig. 1D,E). A comparison of *TM4SF18* expression levels in three pairs of GC tissues and paired GC adjacent tissues using the immunohistochemistry assay revealed the same aforementioned results (Fig. 1F,G). Analysis of the clinicopathological data showed that the expression level of *TM4SF18* correlated with the degree of differentiation, lymph node status, and TNM stage (Table 1). Thereafter, to evaluate the diagnostic efficacy of *TM4SF18* and its ability to determine the prognosis of GC, we evaluated the survival of GC patients in the TCGA database and collected samples in the *TM4SF18* high‐ and low‐expression groups using the Kaplan–Meier curve, and the results showed that the survival of GC patients in the high‐expression group was poorer than in the *TM4SF18* low‐expression group (Fig. 1H,I). When the receiver operating characteristic (ROC) curve was used to analyze the diagnostic profile of *TM4SF18* for GC, the area under the ROC curve was found to be 0.786 (Fig. 1J), suggesting that *TM4SF18* has good diagnostic efficacy. In summary, *TM4SF18* expression is upregulated in GC tissues and has the potential to diagnose and predict GC prognosis.

**Table 1:** Bentley clinical analysis of *TM4SF18*.

Parameter	No. of patients	*TM4SF18* (high)	*TM4SF18* (low)	*P*‐value
Sex				
Male	18	7	11	0.324
Female	22	12	10	
Age (year)				
< 60	15	4	11	0.182
≥ 60	25	12	13	
Tumor size				
< 5	23	13	10	0.337
≥ 5	17	7	10	
Differentiation grade				
Well‐moderate	13	5	8	0.043*
Poor‐undifferentiation	27	20	7	
Organization credit type				
Adenocarcinoma	24	15	9	0.254
Squamous cell carcinomas	9	3	6	
Gland scale cancer	7	3	4	
T stage				
T1–T2	14	7	7	0.168
T3–T4	26	20	6	
Lymph node status				
Positive	25	20	5	0.032*
Negative	15	12	3	
TNM stage				
I–II	22	17	5	0.045*
III–IV	18	16	2	
Nerve/vascular invasion				
Positive	20	15	5	0.311
Negative	20	12	8	

* *P* < 0.05.

### 3.2 *TM4SF18* affects the pathway associated with GC

**Fig. 2:**
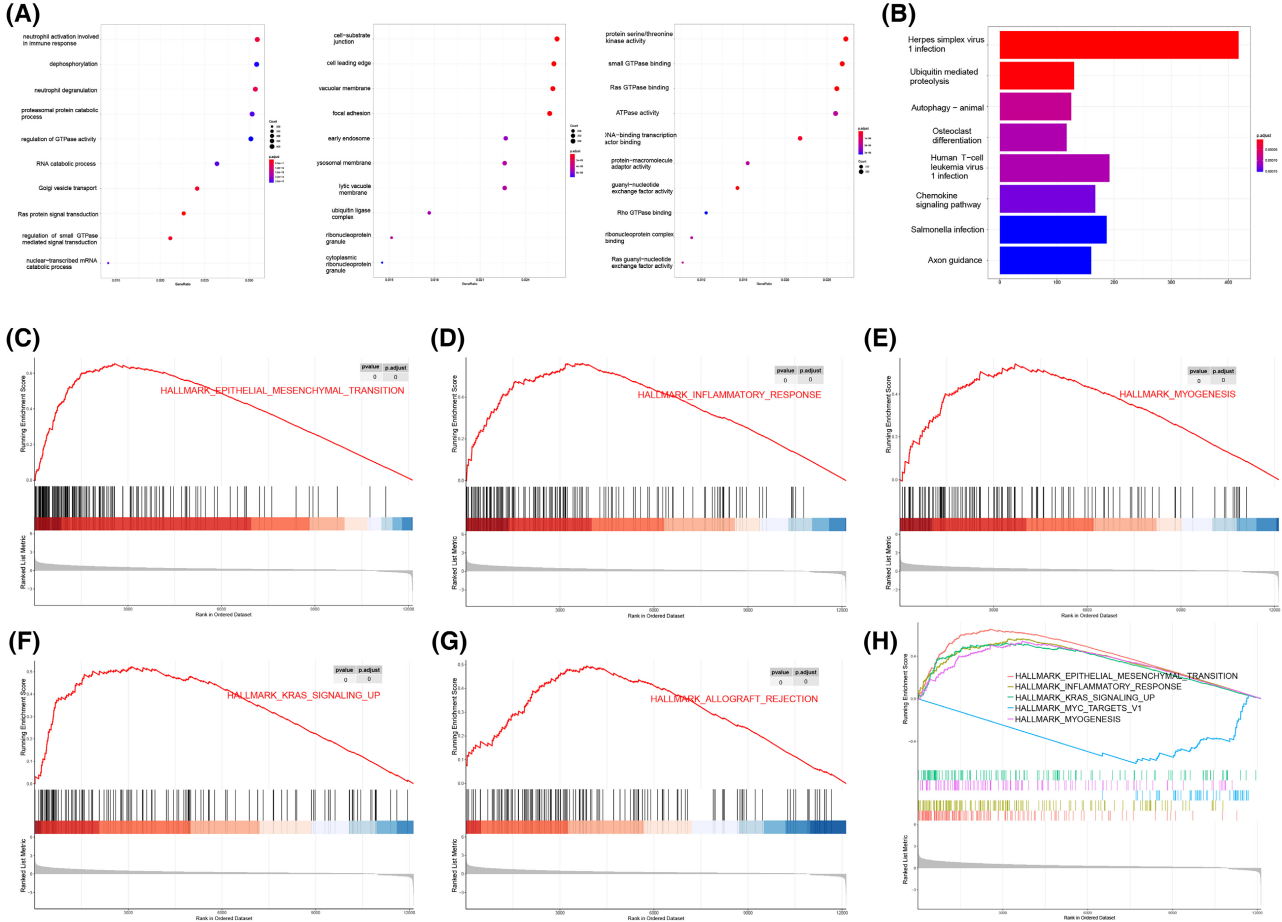
Pathway enrichment analysis of *TM4SF18*. (A) Enrichment analysis of the GO function of *TM4SF18* in 375 GC tissues samples in the TCGA database. (B) KEGG pathway enrichment analysis of *TM4SF18* in 375 GC tissues samples in the TCGA database. (C–H) Enrichment analysis of GSEA gene of *TM4SF18* in 375 GC tissues samples in the TCGA database.

To investigate the pathway of *TM4SF18* in GC, we performed GO function and KEGG pathway enrichment analysis of differentially expressed genes between the high and low TM4SF18 groups and found that the most enriched mRNAs associated with *TM4SF18* in the cellular component process included cell and substrate junction, cell leading edge, and vacuolar membrane. The most significant enrichment of mRNAs associated with *TM4SF18* in the cellular component process included cell‐substrate junction, cell‐leading edge, and vacuolar membrane. The most significant enrichment in the biological process included neutrophil activation involved in immune response; molecular function processes are the most enriched in, for example, protein serine/threonine kinase activity, small GTPase binding, and Ras GTPase binding (Fig. 2A). The pathway enrichment results showed that such genes were significantly enriched in herpes simplex virus 1 infection, endocytosis, human T‐cell leukemia virus 1 infection, and other pathways (Fig. 2B). Furthermore, based on the expression levels of *TM4SF18* in the TCGA database, we used GSEA to verify the classification of its associated biological processes and signaling pathways, and the results showed that the differential expression of *TM4SF18* was associated with numerous biochemical processes in cells, including EMT pathway, inflammatory response pathway, kras signaling up the pathway, myc targets v1 pathway, myogenesis pathway, peroxisome, oxidative phosphorylation antigen processing and presentation, and cytosolic DNA sensing pathway (Fig. 2C–H, Table 2). The aforementioned enrichment results suggest that *TM4SF18* may participate in or influence the biological processes of GC through numerous pathways.

**Table 2:** Enrichment plots from GSEA.

ID	NES	*P*.adjust	*q* Values
HALLMARK_EPITHELIAL_MESENCHYMAL_TRANSITION	2.227353	1.40E‐09	7.37E‐10
HALLMARK_INFLAMMATORY_RESPONSE	1.909581	1.40E‐09	7.37E‐10
HALLMARK_MYC_TARGETS_V1	3.105593	1.40E‐09	7.37E‐10
HALLMARK_MYOGENESIS	1.823724	1.35E‐07	7.11E‐08
HALLMARK_KRAS_SIGNALING_UP	1.767718	1.80E‐07	9.47E‐08

### 3.3 *TM4SF18* promotes the proliferation, migration, and invasion abilities of GC and affects the EMT pathway

**Fig. 3:**
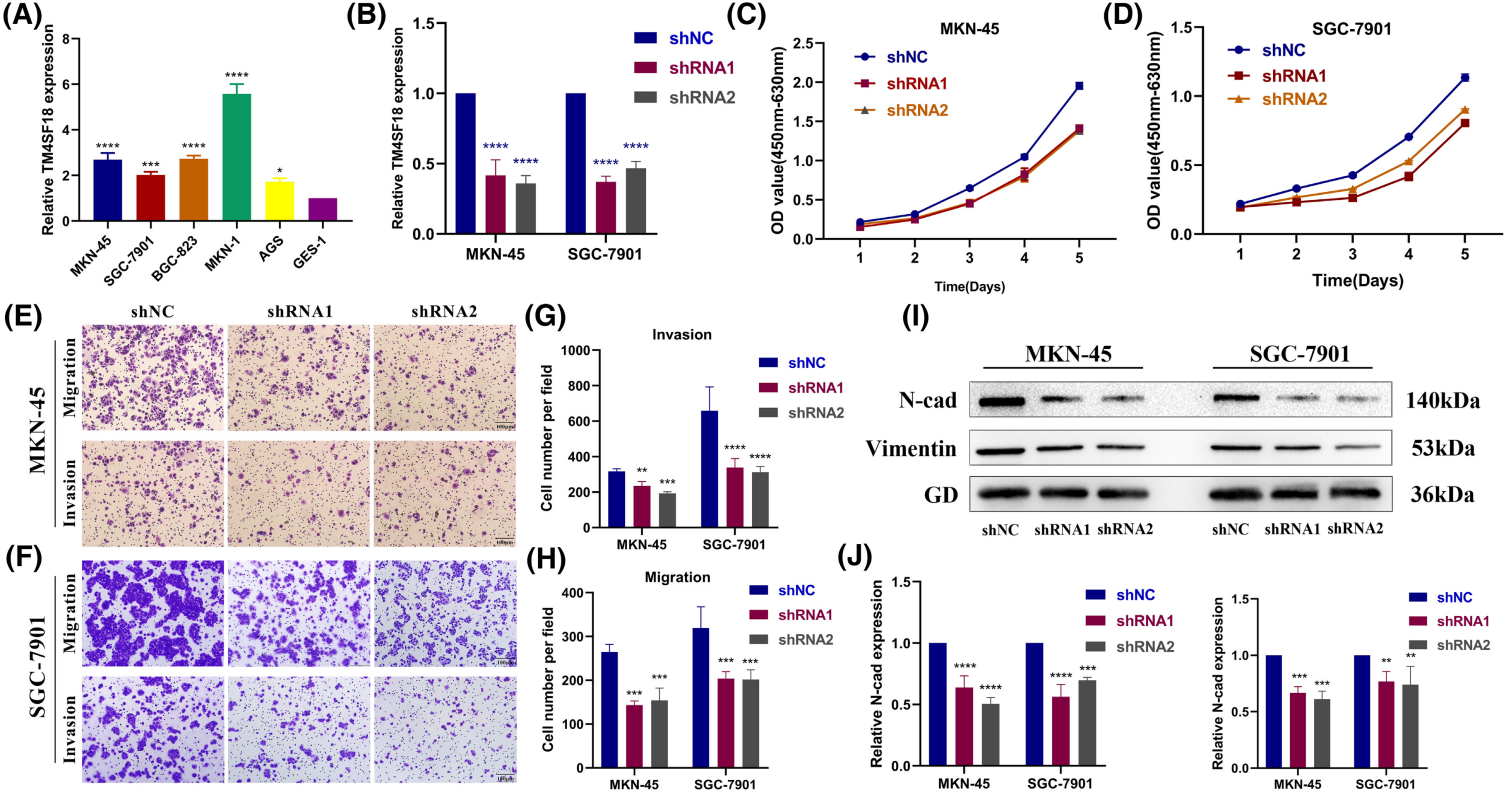
*TM4SF18* promotes the proliferation, migration and invasion of GC cells and affects the EMT process. (A) Expression of *TM4SF18* in GC cells. (B) Knockdown efficiency of *TM4SF18* in MKN‐45 and SGC‐7901 cells. (C, D) CCK‐8 assay to verify the effect of knockdown of *TM4SF18* on the proliferation ability of GC cells. (E–H) Transwell assay to verify the effect of knockdown of *TM4SF18* on migration and invasion of GC cells (magnification ×100). (I, J) Western blot assay and RT‐qPCR assay to verify the effect of knockdown of *TM4SF18* on the expression of EMT key proteins (N‐cad, vimentin) in GC cells. The significance of the results was determined by ANOVA in A, B, G, H, and J. All quantitative data were presented as mean ± SD of three replicates from three independent experiments (scale bar is 100 μm). ****P < 0.0001, ***P < 0.001, **P < 0.01, *P < 0.05.

To investigate the effect of *TM4SF18* on the proliferation, migration, and invasion abilities of GC cells, we performed *in vitro* cellular experiments. We found that the expression levels of *TM4SF18* were all upregulated in GC cells (MKN‐45, SGC‐7901, BGC‐823, MKN‐1, and AGS) (Fig. 3A). Subsequently, we constructed *TM4SF18* knockdown plasmids and transfected them into MKN‐45 and SGC‐7901 cells (Fig. 3B). CCK‐8 assays revealed that knockdown of *TM4SF18* significantly reduced the growth rate of MKN‐45 and SGC‐7901 cells (Fig. 3C,D). Transwell assays showed that knockdown of *TM4SF18* significantly inhibited the migration and invasion abilities of MKN‐45 and SGC‐7901 cells (Fig. 3E–H). In the passageway analysis, we knew that *TM4SF18* might affect GC progression through the EMT pathway. Subsequently, we attempted to explore the effect of *TM4SF18* in GC on the key proteins N‐cad and vimentin in the EMT process. Western blot experiments revealed that the protein levels of N‐cad and vimentin were decreased after the knockdown of *TM4SF18* compared with the controls. The same results were obtained from RT‐qPCR experiments (Fig. 3I,J). In summary, knockdown of *TM4SF18* inhibited proliferation, migration, and invasion of GC cells and affected the expression of key proteins in their EMT.

### 3.4 *TM4SF18* can be an independent factor for the prognosis of GC

**Fig. 4:**
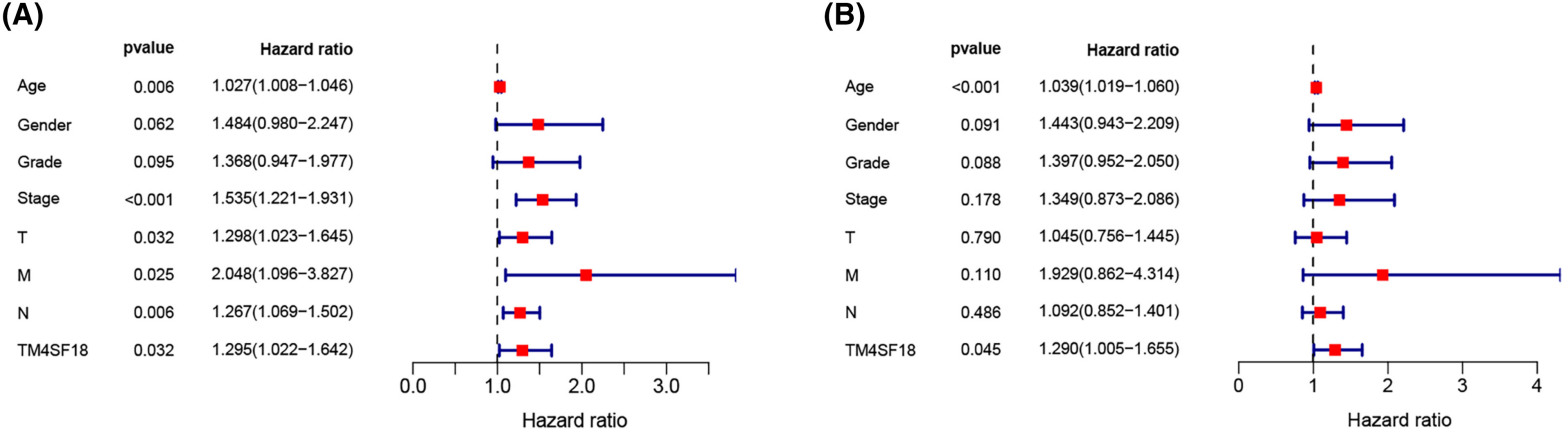
Univariate and multifactor Cox regression analyses of *TM4SF18*. (A) Univariate Cox regression analysis of *TM4SF18* showed that the factors affecting the survival of GC patients included age, gender, grade stage, stage, T‐stage, M‐stage, N‐stage, and *TM4SF18* expression level. (B) Multifactor Cox regression analysis of *TM4SF18* showed that the factors influencing the survival of GC patients included age, gender, grade stage, and *TM4SF18* expression level. All error bars represent CI (95% confidence interval).

To investigate the effect of *TM4SF18* expression level and other clinicopathological features on the survival of GC patients, we performed univariate and multivariate Cox regression analyses on GC patients with complete pathological features in the TCGA database. Univariate Cox regression analysis showed that factors affecting survival in GC included age (*P* = 0.006, HR = 1.027), gender (*P* = 0.062, HR = 1.484), grade stage (*P* = 0.095, HR = 1.368), stage (*P* < 0.001, HR = 1.535), T‐stage (*P* = 0.032, HR = 1.298), M‐stage (*P* = 0.025, HR = 2.048), N‐stage (*P* = 0.006, HR = 1.267), and *TM4SF18* expression levels (*P* = 0.032, HR = 1.295) (Fig. 4A, Table 3). Multivariate Cox regression analysis further revealed that the factors affecting the survival of GC patients included age (*P* < 0.001, HR = 1.039), gender (*P* = 0.091, HR = 1.443), grade stage (*P* = 0.088, HR = 1.397), and *TM4SF18* expression level (*P* = 0.045, HR = 1.290) (Fig. 4B, Table 3). The aforementioned results suggest that *TM4SF18* can be an independent factor to predict the prognosis of GC patients.

**Table 3:** Univariate and multifactor COX regression analysis of the relationship between *TM4SF18* expression and overall survival in patients with GC.

Parameter	Univariate analysis			Multivariate analysis		
	HR	95% CI	*P*‐value	HR	95% CI	*P*‐value
Age	1.027	1.008–1.046	0.006	1.039	1.019–1.060	0.000***
Gender	1.484	0.980–2.247	0.062	1.443	0.943–2.209	0.091
Grade	1.368	0.947–1.977	0.095	1.397	0.952–2.050	0.088
Stage	1.535	1.221–1.931	0.000***	1.349	0.873–2.086	0.178
T	1.298	1.023–1.645	0.032*	1.045	0.756–1.445	0.790
M	2.048	1.096–3.827	0.025*	1.929	0.862–4.314	0.110
N	1.267	1.069–1.502	0.006**	1.092	0.852–1.401	0.486
*TM4SF18*	1.295	1.022–1.642	0.032*	1.290	1.005–1.655	0.045*

***P < 0.001, **P < 0.01, *P < 0.05.

### 3.5 Nomogram plots can effectively predict the prognosis of GC patients

**Fig. 5:**
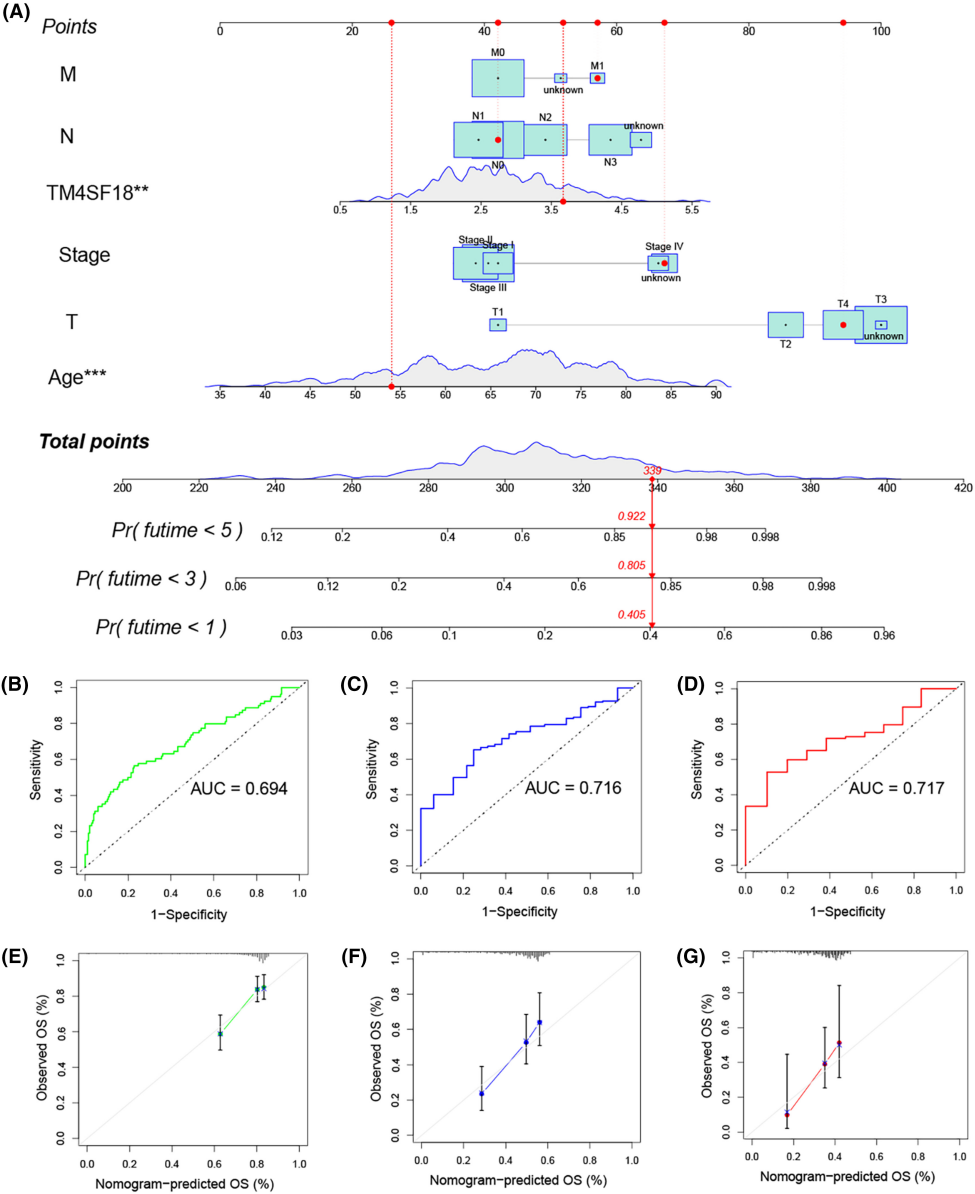
Predicting prognosis of GC patients using nomogram. (A) Nomogram of *TM4SF18* expression predicting overall survival of GC patients. (B–D) ROC curves and calculated area under curve (AUC) in 1‐, 3‐, and 5‐year prognosis of GC patients using nomogram. (E–G) Nomogram corrected plots predicting 1‐, 3‐ and 5‐year survival. Data were obtained from the TCGA database, including 368 GC tissues (patients with missing clinical data were excluded), and the clinical trait data matrix was intersected with the gene expression matrix. All error bars indicate CI (95% confidence interval). ***P < 0.001, **P < 0.01.

To more accurately predict the prognosis of GC patients, we constructed a nomogram containing five clinical characteristics and *TM4SF18* expression levels (Fig. 5A). In this model, we defined a score for each risk factor, established a risk classification system based on the total score obtained by the patient in the model and used the median to select the threshold value. To determine whether the nomogram could better predict the prognosis of GC patients, we plotted time‐dependent ROC curves for OS, with areas under the ROC curves of 0.694, 0.716, and 0.717 for 1‐, 3‐, and 5‐year OS, respectively (Fig. 5B–D). In addition, the calibration curves predicted in the nomogram for 1‐, 3‐, and 5‐year OS do not deviate from the reference line, so their predictions come out with good confidence (Fig. 5E–G). In conclusion, the nomogram we constructed could better predict the prognosis of GC patients.

### 3.6 Correlation analysis of *TM4SF18* expression level and immune infiltration

**Fig. 6:**
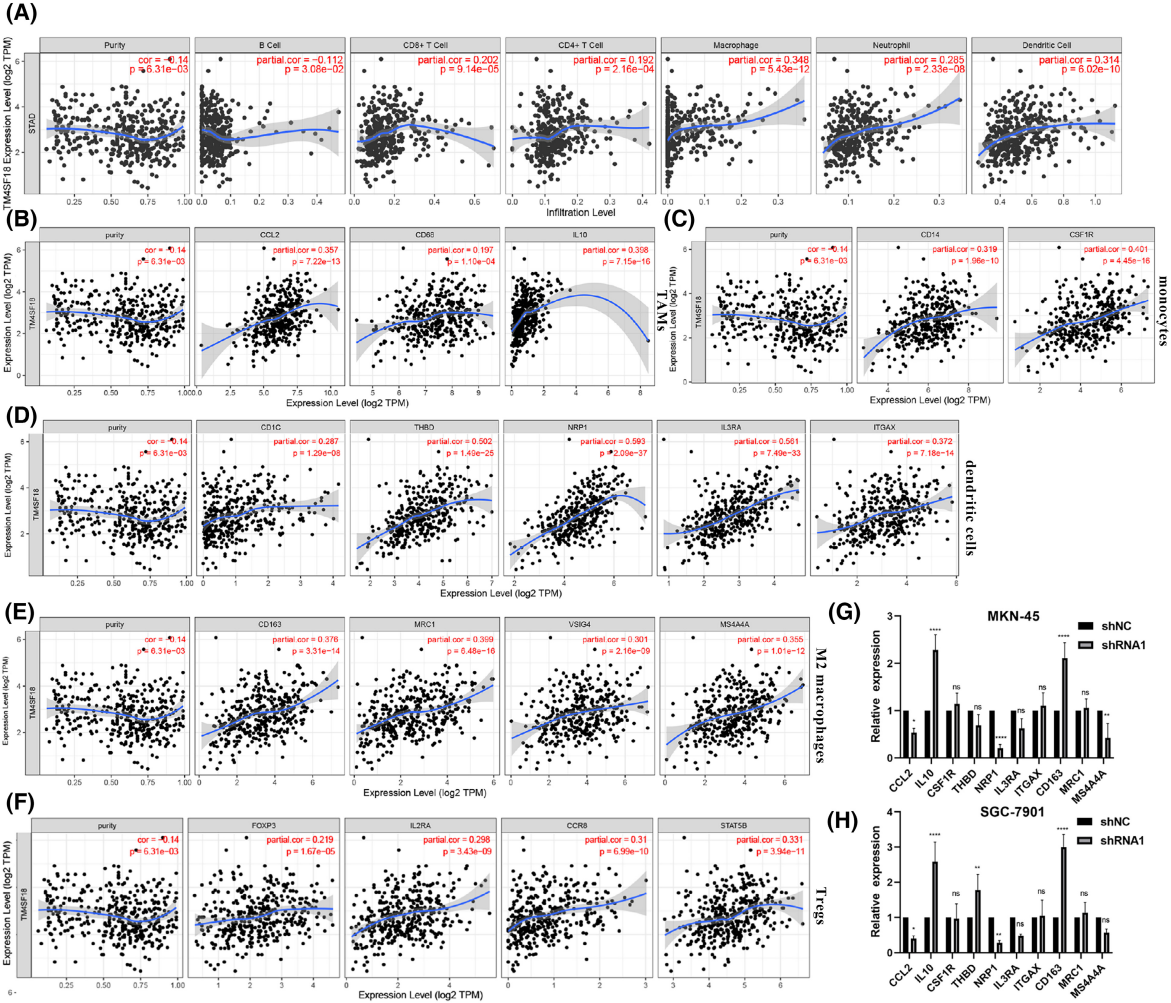
Correlation of *TM4SF18* expression with immune infiltration. Correlation between *TM4SF18* expression and immune cells (A) and most immune marker genes (B–F) in GC. (G, H) Expression of selected immune cell markers after knockdown of *TM4SF18* in MKN‐45 and SGC‐7901 cells. Data represent the mean ± SD of three separate experiments. Analyses of A–F were conducted by TIMER database. The significance of the results was determined by using spearman for A–F and ANOVA for G and H. ****P < 0.0001, **P < 0.01, *P < 0.05.

To investigate the correlation between *TM4SF18* and immune infiltration, we downloaded the immune scores of GC patients from the TIMER database and analyzed them to find the correlation between the expression levels of *TM4SF18* and Macrophage (cor = 0.348, *P* = 5.43e−12), neutrophil (cor = 0.285, *P* = 2.33e−08), and dendritic cell (cor = 0.314, *P* = 6.02e−10) infiltration levels and found them to be positively correlated (Fig. 6A). After adjustment for purity correlation, it was found that the expression level of *TM4SF18* showed a negative correlation with most marker genes of immune cells (Table 4), especially with markers of dendritic cells, M2 macrophages, monocytes, TAMs, and Tregs (Fig. 6B–F). To verify the correlation between *TM4SF18* expression and immune cell infiltration, we verified the expression of 10 immune cell markers with correlation coefficients > 0.35 after the knockdown of *TM4SF18* in MKN‐45 and SGC‐7901 cells. Most of the immune cell markers were found to be meaningfully upregulated or downregulated (the upregulated included markers *IL10*, *THBD*, and *CD163*; the downregulated markers included *CCL2*, *NRP1*, and *MS4A4A*) after knockdown of *TM4SF18* as compared with controls (Fig. 6G,H). The aforementioned results suggest that the expression of *TM4SF18* correlates with the immune infiltration of GC cells.

**Table 4:** Correlation analysis of *TM4SF18* expression with immune cell‐related markers using TIMER database data.

Description	Gene markers	GC			
		None		Purity	
		Cor	*P*	Cor	*P*
CD8^+^ T cell	CD8A	0.175	***	0.149	**
	CD8B	0.117	*	0.102	*
	PTPRC	0.371	****	0.350	****
T cell (general)	CD3D	0.207	****	0.173	***
	CD3E	0.190	***	0.154	**
	CD2	0.244	****	0.214	****
B cell	CD19	0.151	**	0.125	*
	CD79A	0.157	**	0.125	*
	CD27	0.194	***	0.162	**
	KRT20	0.011	ns	0.004	ns
Monocyte	CD14	0.341	****	0.319	****
	CSF1R	0.418	****	0.401	****
TAM	CCL2	0.375	****	0.357	****
	CD68	0.215	****	0.197	***
	IL10	0.417	****	0.398	****
M1 macrophage	NOS2	0.010	ns	−0.003	ns
	CD80	0.349	****	0.330	****
	IRF5	0.124	*	0.110	*
	IL6	0.397	****	0.383	****
	FCGR1A	0.242	****	0.218	****
M2 macrophage	CD163	0.393	****	0.376	****
	MRC1	0.411	****	0.399	****
	VSIG4	0.317	****	0.301	****
	MS4A4A	0.372	****	0.355	****
Neutrophils	CEACAM8	0.129	*	0.133	**
	ITGAM	0.386	****	0.372	****
	FUT4	0.077	ns	0.095	ns
Natural killer cell	KIR2DL1	0.259	****	0.252	****
	KIR2DL3	0.179	***	0.164	**
	KIR3DL1	0.177	***	0.162	**
	KIR3DL2	0.203	****	0.185	***
	NCAM1	0.262	****	0.249	****
	NCR1	0.182	***	0.165	**
Dendritic cell	CD1C	0.312	****	0.287	****
	THBD	0.515	****	0.502	****
	NRP1	0.603	****	0.593	****
	IL3RA	0.573	****	0.561	****
	ITGAX	0.390	****	0.372	****
Th1	TBX21	0.228	****	0.201	****
	STAT4	0.349	****	0.327	****
	STAT1	0.095	ns	0.081	ns
Th2	GATA3	0.164	**	0.143	**
	STAT6	0.139	**	0.142	**
	IL13	0.053	ns	0.053	ns
Tfh	BCL6	0.250	****	0.235	****
	IL21	0.114	*	0.097	ns
Th17	STAT3	0.361	****	0.356	****
	IL17A	0.093	ns	0.077	ns
Treg	FOXP3	0.244	****	0.219	****
	IL2RA	0.318	****	0.298	****
	CCR8	0.326	****	0.310	****
	STAT5B	0.331	****	0.331	****
T cell exhaustion	PDCD1	0.110	*	0.088	ns
	CTLA4	0.218	****	0.196	***
	LAG3	0.113	*	0.085	ns
	HAVCR2	0.319	****	0.296	****

****P < 0.0001, ***P < 0.001, **P < 0.01, *P < 0.05.

### 3.7 Correlation of *TM4SF18* expression with immune cells and immune function

**Fig. 7:**
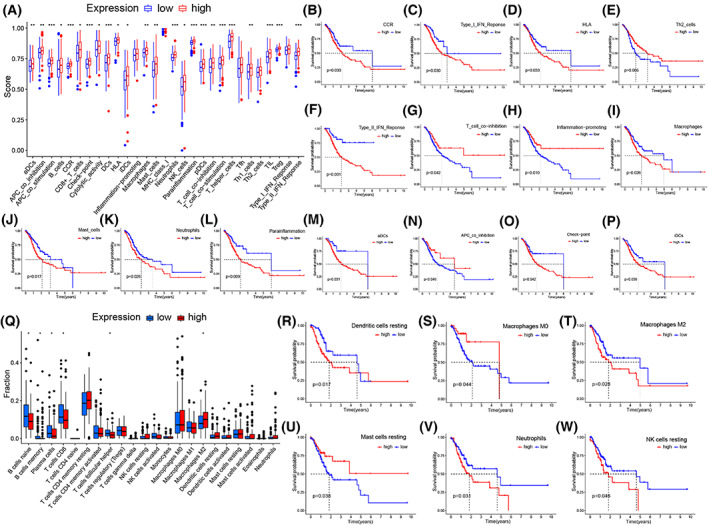
Correlation analysis of *TM4SF18* expression with immune cells and immune function. (A) Differences in *TM4SF18* expression between different immune functions such as aDCs, CCR, and tumor infiltrating lymphocytes (TIL). (B–P) Kaplan–Meier survival analysis of the prognostic value of different immune‐related pathways in GC. (Q) Differential expression of *TM4SF18* in different immune cells. (R–W) Kaplan–Meier survival analysis of the prognostic value of different immune cells in GC. Data were obtained from the TCGA database of 375 GC tissues. Wilcoxon test was used for A and Q, and log rank test was used for the rest to determine the significance of the results. All error bars represent SD. ***P < 0.001, **P < 0.01, *P < 0.05.

To investigate the relationship between *TM4SF18* and tumor‐infiltrating immune cells and functions, we quantified the enrichment fractions of different immune‐related functions and pathways, and cell subpopulations using single‐sample GSEA. In comparing the 29 immune‐related pathways in the *TM4SF18* high‐ and low‐expression groups, activated dendritic cells (aDCs), APC_co_inhibition, chemokine receptor (CCR), Check‐point, HLA, iDCs, Inflammation‐promoting, Macrophages, Mast_cells Neutrophils, Parainflammation, T_cell_co‐inhibition, Th2_cells, Type_I_IFN_Reponse, Type_II_IFN_Reponse, and 24 other pathways had significant differences (*P* < 0.05, Fig. 7A). Thereafter, Kaplan–Meier survival analysis found that among the 29 immune‐related pathways, CCR, Type_I_IFN_Reponse, HLA, Th2_cells, Type_II_IFN_Reponse, T_cell_co‐inhibition, Inflammation‐promoting, Macrophages, Mast_cells, Neutrophils, Parainflammation, and 4 other pathways had a significant survival correlation between the *TM4SF18* high‐ and low‐expression groups (*P* < 0.05, Fig. 7B–P, Table 5). We then analyzed 22 immune cell subsets in tumor immunity using the CIBERSORT method and found that B cells naive, B cells memory, plasma cells, T cells CD8, T cells follicular helper, and macrophages M2 in *TM4SF18* were statistically significant (*P* < 0.05, Fig. 7Q) between the *TM4SF18* high‐ and low‐expression groups. Kaplan–Meier survival analysis found that dendritic cells resting, macrophages M0, macrophages M2, mast cells resting, neutrophils, and NK cells resting had a significant survival correlation between the *TM4SF18* high‐ and low‐expression groups (*P* < 0.05, Fig. 7R–W).

**Table 5:** Differential analysis of *TM4SF18* expression and immune function and functional analysis.

Immune	Cutpoint	*P*‐value
aDCs	0.628	0.031*
APC_co_inhibition	0.858	0.046*
CCR	0.681	0.033*
Check‐point	0.67	0.042*
HLA	0.873	0.033*
iDCs	0.499	0.039*
Inflammation‐promoting	0.832	0.01**
Macrophages	0.784	0.026*
Mast_cells	0.648	0.017*
Neutrophils	0.785	0.026*
Parainflammation	0.865	0.009**
T_cell_co‐inhibition	0.767	0.042*
Th2_cells	0.624	0.006**
Type_I_IFN_Reponse	0.771	0.03*
Type_II_IFN_Reponse	0.73	0.001***

***P < 0.001, **P < 0.01, *P < 0.05.

### 3.8 Correlation between *TM4SF18* expression and tumor microenvironment and immune evasion

**Fig. 8:**
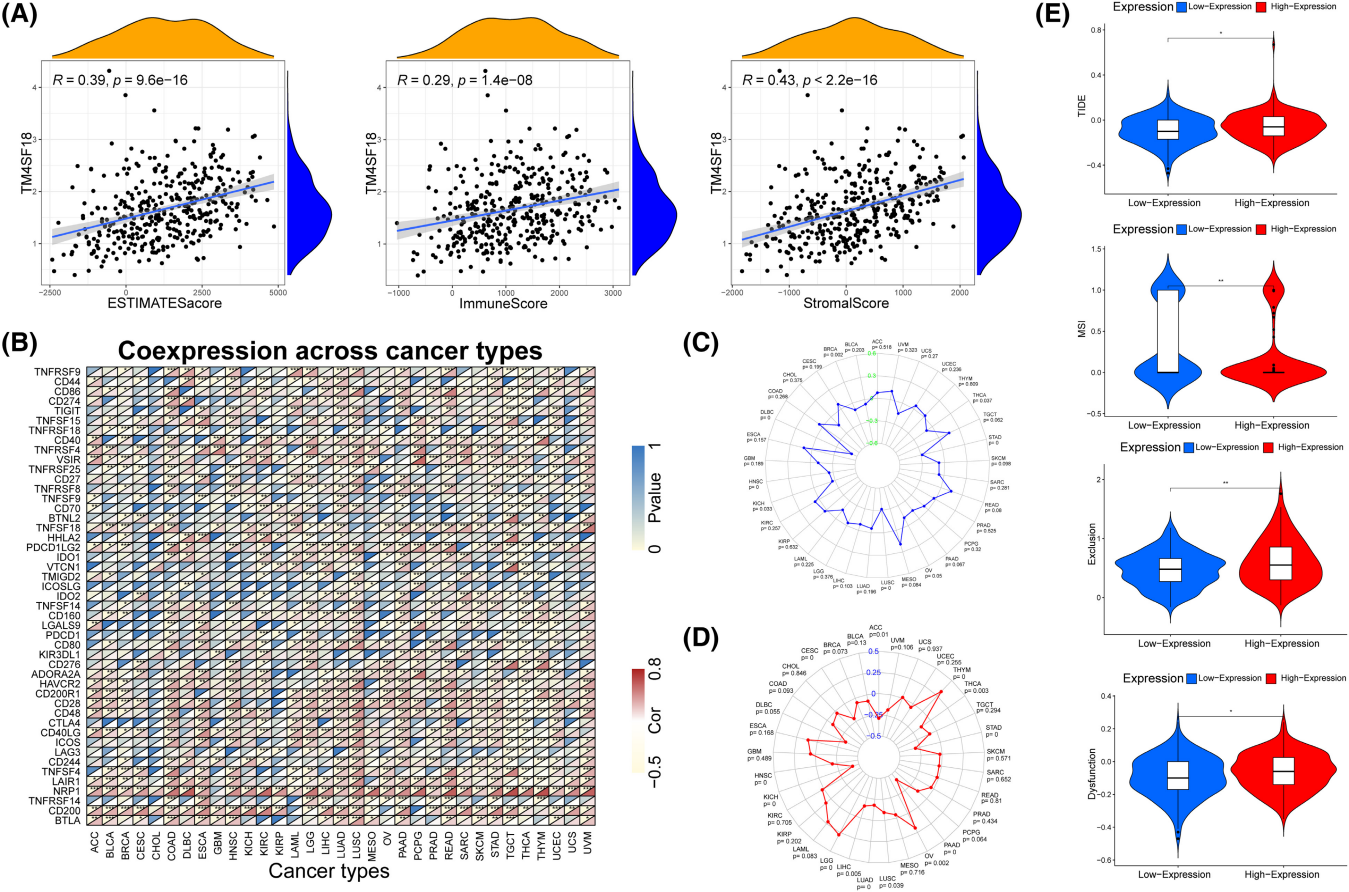
Correlation analysis of *TM4SF18* expression with TME and immune escape. (A) The relationship between *TM4SF18* expression and immune infiltration in GC. (B) Correlation analysis of *TM4SF18* expression levels with common immune checkpoint gene levels in human pan‐cancer tissues. (C, D) Radar plots showing the correlation of *TM4SF18* with TMB (A) and MSI (C) in 33 cancers. Black and blue numbers represent Spearman correlation coefficients. (E) Correlation analysis of *TM4SF18* expression with TIDE. Data in the figure, where the analytical data for A‐D were obtained from the UCSC Xena website (*N* = 10 535) and the analytical data for E were obtained from the TCGA database including 375 GC tissues. Spearman‐test was used for A and pearson chi‐square fitting test was used for B to determine the significance of the results. ***P < 0.001, **P < 0.01, *P < 0.05.

There is now evidence that the TME has an important role in tumorigenesis and progression. TME stimulates tumor cells and causes heterogeneity, which contributes to enhanced drug resistance in tumor cells and further accelerates the progression of GC [17]. We evaluated the ESTIMATESScore, ImmuneScore, and StromalScore scores of GC in the TCGA database using an estimation algorithm and analyzed the correlation between the expression level of *TM4SF18* and these three scores. The results showed that the expression level of *TM4SF18* in GC was positively correlated with the three scores (Fig. 8A). The correlation analysis between *TM4SF18* and checkpoint gene expression showed that *TNFRSF9*, *CD44*, *CD86*, *TNFSF15*, *CD40*, *TNFRSF4*, *VSIR*, *TNFESF8*, *PDCD1LG2*, *TNFSF14*, *CD80*, *CD276*, *HAVCR2*, *CD28*, and *CD48* in GC were highly correlated in expression (Fig. 8B). We investigated the relationship between TMB and microsatellite instability (MSI) and *TM4SF18* expression in different tumor types expressed, and the results showed that the expression of *TM4SF18* in breast invasive carcinoma, lymphoid neoplasm diffuse large B‐cell lymphoma, HNSC, KICH, LUSC, ovarian serous cystadenocarcinoma, and STAD was significantly negatively correlated with MSI (*P* < 0.05). The correlation coefficient between lymphoid neoplasm diffuse large B‐cell lymphoma and STAD was the highest (Fig. 8C). After we analyzed the correlation between *TM4SF18* expression and TMB in different tumors, we found that *TM4SF18* expression in adrenocortical carcinoma, cervical squamous cell carcinoma and endocervical adenocarcinoma, HNSC, KICH, brain lower‐grade glioma, liver hepatocellular carcinoma, lung adenocarcinoma, LUSC, ovarian serous cystadenocarcinoma, pancreatic adenocarcinoma, STAD, thyroid carcinoma, and thymoma was significantly correlated with TMB (*P* < 0.05) (Fig. 8D). Thereafter, we assessed the potential clinical efficacy of immunotherapy with a different *TM4SF18* using TIDE. The higher the predicted score of TIDE, the higher the likelihood of immune evasion, which indicates that patients are less likely to benefit from immune checkpoint inhibitor (ICI) therapy. Our results showed that patients with high *TM4SF18* expression had a higher TIDE prediction score than the *TM4SF18* low‐expression group (Fig. 8E). This also demonstrates that patients with low expression benefit more from ICI treatment relative to the patients with high *TM4SF18* expression. In addition, higher TIDE scores indicated that patients had a worse prognosis. Therefore, our results also suggest that the *TM4SF18* low‐expression group with a low TIDE prediction score may have a better prognosis relative to the *TM4SF18* high‐expression group with a high TIDE prediction. In addition, we found that the *TM4SF18* high‐expression group had significantly different MSI scores, higher T‐cell rejection scores and T‐cell dysfunction scores (Fig. 8E). All of these results suggest that *TM4SF18* is associated with TME and that patients with high *TM4SF18* expression may have poor ICI treatment and poorer prognosis.

## 4 Discussion

Gastric cancer is one of the most common tumors with multiple predisposing causes, a high mortality rate, and a lack of effective diagnostic and therapeutic options. Therefore, there is an urgent need to find effective diagnostic and prognostic biomarkers in GC. In recent years, advances in genetic analysis have led to the identification of numerous key genes, the majority of which are used as biomarkers or therapeutic targets for tumors. Among them is the L6 family of proteins. This family has been found to have multiple roles in tumor development, and family member *TM4SF1* has been reported to play an important role in a variety of tumors including lung [18], prostate [9], breast [19], liver [20, 21], bladder [22], and pancreatic cancers [11, 13, 23]. *TM4SF4* and *TM4SF5* in the L6 family are also involved in tumor regulation, but their exact mechanism is not yet known [24]. In addition, *TM4SF18* is the only L6 family protein in the family known to promote tumor cell growth. It was found that *TM4SF18* shares 60% amino acid sequence homology with *TM4SF1*, but the mechanism of action remains unclear [12, 23]. We analyzed data from the TCGA database, performed RT‐qPCR experiments, and found that *TM4SF18* expression levels were significantly upregulated in GC tissues. The ROC curve and Kaplan–Meier risk estimation method demonstrated that *TM4SF18* can be used as a biomarker for GC and can be used to monitor the prognosis of GC dynamically. *In vitro* experiments revealed that *TM4SF18* promoted the proliferation, migration, and invasion abilities of GC cells. This corroborates previous reports in the literature. *TM4SF18* was found to be involved in GC tumor progression through the EMT pathway by GSEA enrichment analysis. Thereafter, knockdown of *TM4SF18* was found to effectively reduce the expression of key EMT proteins N‐cad and vimentin protein and RNA levels by western blot and RT‐qPCR experiments. This also suggests that *TM4SF18* may regulate the biological process of GC through the EMT pathway. Furthermore, with the use of univariate and multifactorial Cox regression analyses and nomogram construction, *TM4SF18* was found to be a prognostic independent factor in GC and could effectively predict the prognosis of GC patients.

Tumor progression is determined by the cellular profile and influenced by TME, which is the internal environment for malignant tumor progression. TME reduces tumor cell resistance to chemotherapy and immunotherapy [17]. It has been found that EMT is a major regulator of tumor metastasis and may be involved in the interaction between tumor cells and TME [25, 26, 27]. In our study, *TM4SF18* was found to be associated with both immune infiltration and TME. With the use of the TIMER database, the expression level of *TM4SF18* was found to be positively correlated with the degree of infiltration of lymphocytes, neutrophils, dendritic cells, and most marker genes. It has been shown that Tregs of tumor patient origin usually express different CCRs, which contribute to their migration into the tumor in response to signals sent by TME [28]. It has also been suggested that Treg infiltration is prognostically beneficial in patients with GC. Li et al. [29]found that infiltration of GARP^+^ Tregs may serve as a novel prognostic factor for GC response to neoadjuvant chemotherapy. Thus, infiltration of Tregs is a double‐edged sword in the progression of GC. Furthermore, quantification of different immune‐related pathways and cell subpopulation enrichment scores using single‐sample GSEA as well as CIBERSORT revealed a significant correlation between *TM4SF18* expression and numerous immune‐related pathways such as T_cell_co‐inhibition and numerous immune cell subpopulations such as T cells CD8. This further corroborates the possibility of the hypothesis of *TM4SF18* as a central gene. TME includes not only the above but also TMB, MSI, and immune checkpoints. TMB is an independent biomarker that has been discovered in recent years in a variety of tumor immunotherapies and can be used to predict the efficacy of immunotherapy [30, 31]. Patients with high TMB expression have been shown to benefit more from ICI therapy [32]. As the number of gene variants increases, more new antigens are created, and the more likely the immune system recognizes them. Additionally, MSI is now considered to be an indicator to distinguish between numerous tumor types [33]. Our study found a significant correlation between *TM4SF18* expression and TMB and MSI in a variety of tumors. Furthermore, in terms of treatment, ICI therapy is effective in treating recurrence [23, 34, 35]. Currently, the overall response rate of ICI therapy remains low [36, 37]. Hence, being able to obtain greater results from ICI is crucial for a patient. TIDE is an innovative computational approach that can be used to identify factors underlying two mechanisms of tumor immune escape: induction of T‐cell dysfunction in tumors with high infiltration of cytotoxic T lymphocytes and prevention of T‐cell infiltration in tumors with low cytotoxic T lymphocyte levels. Our study found that the *TM4SF18* high‐expression group had a higher predicted score for TIDE, implying the ability to obtain lower treatment outcomes and prognosis from ICI.

## 5 Conclusions

In summary, we found that high expression of *TM4SF18* promotes GC cell proliferation, migration, and invasion and affected the EMT process in GC. Furthermore, *TM4SF18* could affect the immune infiltration and TME of GC in multiple ways and correlates with numerous immune markers. Also, *TM4SF18* could be used as an independent prognostic indicator for dynamic monitoring of GC prognosis. This also provides new options for clinical molecular and immunotherapy.

Although our study shows promise, there are several limitations to the current study. First, most of our study was from public databases and the GC sample collected was small, and we will expand the sample size to continue the prospective study in the future. Second, our study did not include cases of neoadjuvant chemotherapy or radiotherapy for analysis, and the exploration of *TM4SF18* function was not well developed.

## Conflict of interest

The authors declare no conflict of interest.

## Author contributions

XQ and SM drafted the paper, designed the experiment, and selected the topic. XQ and YC were involved in the experimental supplementary part of the whole manuscript and analyzed the data. XQ, YC and SM participated in the revision of the paper. LS participated in data collection and the revision of the manuscript. SJ designed this study & provided resources and guidance for the paper. All authors read and approved the final manuscript.

## Data Availability

The data that support the findings of this study are available from the corresponding jsq_jyk@ntu.edu.cn upon reasonable request.
